# Editorial: Physiological regulation during the full supply chain of fruit and vegetables: main challenges from plant growth to product sales

**DOI:** 10.3389/fpls.2025.1758284

**Published:** 2025-12-16

**Authors:** Yanpei Chen, Dong Li

**Affiliations:** 1 College of Biosystems Engineering and Food Science, Zhejiang University, Hangzhou, China; 2 Innovation Center for Postharvest Agro-Products Technology, Laboratory of Agro-Products Postharvest Handling of Ministry of Agriculture and Rural Affairs, Zhejiang University, Hangzhou, China; 3 The Rural Development Academy, Zhejiang University, Hangzhou, China

**Keywords:** anthocyanin biosynthesis, cell wall polysaccharides, isoamyl isothiocyanate, peptide hormones, photoperiod-potassium interaction, postharvest physiology, synergistic hormone treatment

The global fruit and vegetable industry faces the immense challenge of delivering high-quality, nutritious, and safe produce from farm to fork. Physiological deterioration, driven by ripening, senescence, water loss, and pathogen attack, leads to significant postharvest losses, undermining food security and economic sustainability. A deep understanding of the physiological and molecular mechanisms governing quality attributes—from pre-harvest growth to postharvest storage, is paramount for developing targeted, effective, and sustainable preservation technologies. This Research Topic, “*Physiological Regulation during the Full Supply Chain of Fruit and Vegetables: Main Challenges from Plant Growth to Product Sales,”* brings together a collection of six original research articles and one review that dissect these complex processes and present innovative strategies for quality maintenance.

The journey of quality preservation begins even before harvest. Wang et al. demonstrate that pre-harvest cultivation practices can prime vegetables for extended shelf life. Their work on fresh-cut lettuce reveals a synergistic effect between photoperiod and foliar potassium application on storage tolerance and taste. A 14-hour photoperiod with 0.3% potassium was optimal, enhancing water retention, antioxidant stability, and reducing decay, highlighting how agronomic management can be tailored to improve postharvest performance.

At the genetic level, varietal differences fundamentally dictate a commodity’s physiological potential. Hasan et al. provide a molecular explanation for the higher anthocyanin content in black versus blue barley. Through transcriptomics, they identified thousands of differentially expressed genes and pinpointed key enzymes in the flavonoid pathway, such as ANS1 and LDOX genes, whose elevated expression drives greater anthocyanin biosynthesis and accumulation in black barley. This work offers a genetic roadmap for breeding nutrient-dense crops. Similarly, Zhao et al. investigated the physiological basis of peelability, a key convenience trait in table grapes. They established that easy peeling is negatively correlated with cell wall polysaccharide content (notably protopectin) and positively correlated with the activity of a suite of cell wall-degrading enzymes (e.g., polygalacturonase, pectate lyase). Their comparative study across twelve varieties provides a physiological framework for selecting and breeding grapes with desirable postharvest handling traits.

Once harvested, the battle against senescence intensifies, requiring effective and safe postharvest interventions. This Research Topic presents several advances in this area. He et al. optimized a synergistic postharvest hormone treatment (PEHC) for kiwifruit, combining brassinolide, melatonin, methyl jasmonate, and salicylic acid. This formulation markedly reduced softening and decay over 80 days of cold storage. Integrated transcriptomic and metabolomic analyses revealed that PEHC acts by suppressing ethylene biosynthesis genes and altering metabolite profiles, thereby delaying ripening and preserving quality. Extending beyond higher plants, Dong et al. explored the preservation of matsutake mushroom using isoamyl isothiocyanate (IAITC). They found that a 10 μL L^−1^ IAITC fumigation effectively reduced water loss and browning by bolstering the mushroom’s antioxidant system (e.g., enhancing SOD, CAT activity) and suppressing oxidative damage, showcasing a natural volatile compound’s potential for postharvest management of highly perishable fungi.

Looking to the future, the review by Niu et al. synthesizes the promising horizon of using peptide hormones for postharvest preservation. Positioned as a biodegradable and highly specific alternative to conventional methods, peptide hormones like PSK and CTG134 can delay ripening in peaches and broccoli and alleviate chilling injury in loquats. The authors compellingly argue that emerging technologies, including artificial intelligence and synthetic biology, are poised to overcome current hurdles of cost and stability, paving the way for next-generation, sustainable preservation strategies.

Collectively, the studies in this Research Topic illuminate the continuum of physiological regulation across the supply chain. They underscore that quality is not merely preserved at the end but is built through pre-harvest practices, encoded in genetic makeup, and maintained through targeted postharvest treatments that modulate fundamental physiological pathways. Future research should focus on the translational application of these findings, scaling up promising treatments like PEHC and IAITC, validating genetic markers for breeding programs, and developing integrated management strategies that combine these novel approaches for synergistic effects.

On behalf of the editors of this Research Topic, I extend our sincere gratitude to all the authors for their valuable contributions, to the reviewers for their rigorous and constructive feedback, and to the editorial team for their unwavering support. We believe this Research Topic provides a meaningful advancement in our understanding of horticultural produce physiology and offers practical insights for reducing losses and enhancing quality throughout the global supply chain.

